# Comparison of web-based information about cell-free DNA prenatal screening: implications for differences of sex development care

**DOI:** 10.3389/fruro.2023.1144618

**Published:** 2023-10-31

**Authors:** Soojin Kim, Esther L. Finney, Ushasi Naha, Ilina Rosoklija, Kyle S. Honegger, Allison Goetsch Weisman, Jane L. Holl, Courtney Finlayson, Diane Chen, Emilie K. Johnson

**Affiliations:** ^1^ Department of Urologic Sciences, University of British Columbia, Vancouver, BC, Canada; ^2^ Department of Surgery, Division of Urology, British Columbia Children’s Hospital, Vancouver, BC, Canada; ^3^ Department of Urology, Lahey Hospital & Medical Center, Burlington, MA, United States; ^4^ Department of Urology, Loyola University, Chicago, IL, United States; ^5^ Department of Surgery, Division of Urology, Ann & Robert H. Lurie Children’s Hospital of Chicago, Chicago, IL, United States; ^6^ Data Analytics and Reporting, Ann & Robert H. Lurie Children’s Hospital of Chicago, Chicago, IL, United States; ^7^ Division of Genetics, Genomics and Metabolism, Ann & Robert H. Lurie Children’s Hospital of Chicago, Chicago, IL, United States; ^8^ Department of Pediatrics, Northwestern University Feinberg School of Medicine, Chicago, IL, United States; ^9^ Department of Neurology, Biological Sciences Division, University of Chicago, Chicago, IL, United States; ^10^ Department of Pediatrics, Division of Endocrinology, Ann & Robert H. Lurie Children’s Hospital of Chicago, Chicago, IL, United States; ^11^ Potocsnak Family Division of Adolescent and Young Adult Medicine, Ann & Robert H. Lurie Children’s Hospital of Chicago, Chicago, IL, United States; ^12^ Pritzker Department of Psychiatry and Behavioral Health, Ann & Robert H. Lurie Children’s Hospital of Chicago, Chicago, IL, United States; ^13^ Department of Psychiatry and Behavioral Science, Northwestern University Feinberg School of Medicine, Chicago, IL, United States; ^14^ Department of Urology, Northwestern University Feinberg School of Medicine, Chicago, IL, United States

**Keywords:** cell-free DNA (cfDNA), noninvasive prenatal test (NIPT), web-based information, patient education, fetal sex determination, disorder of sex development, sex chromosome aneuploidies, counseling

## Abstract

**Objective:**

Cell-free DNA (cfDNA) prenatal screening is a commercially available noninvasive test that detects fetal genetic material in maternal blood. While expectant parents often use it for “gender” determination, there is little information about unintended consequences of testing, such as revelation of a difference of sex development (DSD). The study aimed to characterize currently available website information about cfDNA and compare the cfDNA-related content.

**Methods:**

A systematic search for websites with information about cfDNA was conducted using search terms generated by a natural language processing analysis of the results of an Amazon Mechanical Turk (MTurk) survey of 1,000 parents and then performing a “Google” search, using the terms. Commercial cfDNA testing companies (CC) websites were also identified by consulting a genetic counselor (AGW). Data were collected on about each website’s characteristics and information about cfDNA. Information about cfDNA was compared between websites. Data were analyzed using descriptive statistics, Fisher’s exact test or Kruskal-Wallis test were applied, as appropriate.

**Results:**

Sixty websites were identified. After eliminating duplicates, 11 commercial company (CC) websites were identified. Nineteen other websites were reviewed of which six overlapped with five CC websites. Most of the websites had non-professional authors (73.7%), such as laypersons and CC representatives. CC websites were significantly more likely than search term-identified websites to state that cfDNA can screen for trisomy 21 (*p*=0.002), trisomy 18 (*p*<0.0001), trisomy 13 (*p*<0.001), sex chromosome aneuploidies (*p*<0.001), and microdeletions (*p=*0.002).

**Conclusions:**

This study shows that most website currently available information for expectant parents about cfDNA prenatal screening is produced by non-professional organizations. There are significant differences between the information provided by CC and Google search websites, specifically about the number of conditions screened for by cfDNA. Improving availability and quality of information about cfDNA could improve counseling future expectant parents. Inclusion of information about the potential for detection of a DSD is needed.

## 1 Introduction

Cell-free DNA (cfDNA) cfDNA is fetal DNA which is present as free-floating fragments in maternal blood and is thought to come from placental trophoblasts (1, 2). cfDNA can be detected in maternal circulation as early as at 5 weeks gestation and counted and sequenced by means of real time quantitative polymerase chain reaction (qPCR) (3). Detection of cfDNA in maternal blood is also known as a noninvasive prenatal test (NIPT) and used to screen for chromosomal disorders including trisomy 21. Another primary reason for cfDNA testing is to screen for fetal sex chromosome aneuploidy but it is often used for fetal sex determination by parents. Advantages of cfDNA testing include the potential for early detection of fetal abnormalities compared to other standard screening methods, in addition to the noninvasive nature of the test (2–10).

However, there are also disadvantages associated with using cfDNA. cfDNA prenatal screening is not a diagnostic test. Thus, any result indicating an increased risk for a chromosomal abnormality requires confirmation with prenatal or postnatal diagnostic genetic testing. cfDNA screening can also result in a false positive or a false negative result. For example, false positive rate for detecting trisomy 21 can be as low as 0.04%, while it is 3.5 times higher for monosomy X (11). Additionally, results of cfDNA can be discordant with the results of ultrasound (US) or other screening tests. Discordances can occur for a variety of reasons (12), for example, in differences of sex development (DSD) the genotype information based on the cfDNA may not align with the phenotype information obtained on an US (13). Other potential reasons for discordant results include confined placental mosaicism, vanishing twin, maternal karyotype abnormality, and maternal malignancy (12). Occasionally, some genetic findings detected by cfDNA do not lead to any clinical manifestations, hence, would not influence the affected individual at all (12, 13). Expectant parents are often unprepared for the implications of the results and, as a result, may experience a significant levels of anxiety, distress and grief, sometimes unnecessarily (13, 14). Furthermore ethical concerns from potential abuse of this test have raised by bioethicists as well, such as sex selection (15, 16).

In the United States, the cfDNA test did not require approval by the US Food and Drug Administration (FDA) because fetal sex determination is not considered to be a medical diagnosis (15, 17, 18). As a result, a cfDNA prenatal screening technology company promoted the test in the lay media as “the most accurate DNA gender test” with “unprecedented and unsurpassed accuracy” (16, 17) and the test became readily available to expectant parents without any local, federal, or medical professional regulation (19, 20). While there was a rapid increase in the number of women using cfDNA for “gender” determination, there were also increasing reports about misdiagnoses for both “gender” and chromosomal abnormalities, usually in the lay media, which eventually led to a class-action lawsuit against the company (16, 17, 21, 22). However, such direct-to-consumer marketing and test availability by a commercial company without any scientific validation or guidelines for use and counseling are unusual. Although the sources, quality, and delivery of information about cfDNA have been shown to contribute to expectant parents’ anxiety about prenatal diagnosis (14), there is no comprehensive report about the sources, content, and quality of information available to and likely accessed by expectant parents regarding cfDNA. Considering that the lay public accesses most information about cfDNA on websites that are non-professional sources (14, 23), it is imperative to better understand the sources, content, and quality of the information. This study was designed to (1) characterize the sources of information about cfDNA on websites and (2) compare the content of information by web page types. We hypothesize that most websites resulting from an internet search will be created and authored by laypeople and that commercial company websites will be more likely to state the benefits of cfDNA and promote cfDNA to screen for a wider range of conditions than other websites.

## 2 Materials and methods

A systematic search of the World-Wide Web (the Web) for websites with information about cfDNA was conducted, with a focus on information related to fetal sex determination. The search focused on websites with information in English. Websites with information in graphical, pictorial, video, blogs, or tabular formats only were excluded.

### 2.1 Search strategy

Two types of searches of the Web were conducted, using the search engine “Google.” Google was chosen as the search engine because it is currently the most commonly used search engine (80.6% among desktop and 97.1% among mobile/tablet searches (24).

The first search focused on identifying commercial cfDNA testing companies (CC) websites. Identified CC websites were then reviewed for completeness and accuracy by a genetic counselor (AGW). The second search used terms identified by the lay public.

Terms for the second search were identified by an Amazon Mechanical Turk (MTurk) Survey, in April 2019, completed by with 1,000 expectant and recent parents (within the past 12 months), aged ≥18 years, English speaking and located in the United States. MTurk is a crowdsourcing platform which offers access to a diverse group of pay-per-task survey takers. Open-ended questions asking what search terms they would use to learn about blood tests that can find out “if their baby is a boy or a girl” were used. Free text answers were then analyzed using natural language processing to identify relevant four-word phrases that were reported ≥10 times by survey respondents. From these phases, six search terms were constructed, including “baby gender blood test”, “boy girl blood test”, “blood test determine gender”, “gender reveal blood test”, “blood test find gender”, and “blood test sex baby.” A Google search, based on each of these six terms, was performed on a public computer in October of 2019. The cache/cookies were cleared after each search. The top 10 websites, generated from each search term, were included in the analysis. Less than one-third of searches yielded more than 10 websites (25). Websites were re-accessed in November of 2022 to determine the date of last update.

### 2.2 Data elements collected

For each website, the web page type, author, days since the last update, word count, number of graphics and tables, presence of advertisements, and the actual information on cfDNA were recorded. Type was classified as DNA testing lab, home DNA kit, parenting website, government website, non-medical testing facility, or non-profit academic health system. Author was defined as professional (urology, pediatric/other surgery, endocrinology, maternal-fetal-medicine (MFM), genetic, perinatal/neonatology, obstetrics and gynecology, primary care physician, pediatric, or women’s health service), layperson, commercial representative, unspecified, or other (e.g., MPH, PhD and anesthesiologist/intensive care specialist).

Detailed information on the following topics was extracted: ancillary resources (e.g., links to external resources, such as videos, articles, brochures); explanation of cfDNA test mechanism; conditions screened by cfDNA (e.g., trisomy 21, 18 and 13, sex chromosome aneuploidies (SCA), microdeletions); cfDNA use for fetal sex determination; ease of interpretation of cfDNA test result by the lay public consumer; validity, sensitivity and specificity of cfDNA, in general, and for sex determination; application for a multiple pregnancy; limitations, benefits, and risks of cfDNA testing; guidance for positive or negative test results; and resources for genetic counseling. Availability of information was assessed but readability, understandability, accuracy, completeness or quality of information were not evaluated.

Data were included for analysis when present within the website, including subsections or pages accessed by clicking on links or buttons. However, data present in external websites, articles, or from other sources, were not included in the analysis.

### 2.3 Data analysis

Descriptive statistics were used to characterize cfDNA information by website type and content. Univariable analyses were performed, Fisher’s exact test or Kruskal-Wallis test as appropriate, to compare informational content between CC websites and search term-identified websites. Statistical analysis was performed using Stata IC (version 15, College Station, TX). All tests were two-sided and *p* < 0.05 was considered statistically significant.

## 3 Results

A total of 11 CC websites were identified. The search using the six search terms identified by MTurk Survey yielded 60 websites (henceforth, “search term-identified websites”), which after removing duplicates, generated 19 websites for review, six of which were CC sites that overlapped with five of the previously identified CC websites; two websites were the same company with different names in two different countries. Therefore, a total of 13 search term-identified websites were included for data extraction.

Website characteristics are described in **Table 1**. The most common type of websites originated from DNA testing labs certified by Clinical Laboratory Improvement Amendments (CLIA) that offer cfDNA prenatal screening through qualified medical providers (36.8%). Most websites had non-professional authors (73.7%), such as laypersons and CC representatives, and 37% had not been updated in ≥2 years.

**Table 1:** Characteristics of websites (N=19).

Content	No. (%)
Web Page Type (%)	
DNA testing lab	7 (36.8)
Home DNA kit	4 (21.1)
Parenting website	3 (15.8)
Government website	2 (10.5)
Non-medical testing facility	2 (10.5)
Non-profit academic health system	1 (5.3)
Author (%)	
Commercial company	13 (68.4)
Layperson	1 (5.3)
Geneticist	1 (5.3)
MFM* or Layperson	1 (5.3)
Unspecified	1 (5.3)
Other**	2 (10.5)
Year of Last Update	
2022	12 (63.2)
2021	0 (0)
2020 or earlier	7 (36.8)
Word Count (median, range)	728 (148-1532)
Number of Graphics	
0	15 (78.9)
1	4 (21.1)
Number of Tables	
0	15(78.9)
1	4 (21.1)
Presence of Advertisements	
Yes	6 (31.6)
No	13 (68.4)

*MFM: maternal-fetal-medicine; **Other: MPH, PhD (1), Anesthesiologist/Intensive Care Specialist (1).

CI: 95% confidence interval.

**Table 2** shows the availability and content of information on cfDNA on CC and search term-identified websites. CC websites were significantly more likely to report that cfDNA screens for trisomy 21 (*p*=0.002), trisomy 18 (*p*<0.001), trisomy 13 (*p*<0.001), sex chromosome aneuploidies (*p*<0.001), and microdeletions (*p=*0.002) than search term-identified websites. CC compared to search term-identified websites were more likely to support their statement by providing test sensitivity and specificity (*p* =0.017) and outlined benefits (*p*=0.013) and risks (*p*<0.001). Finally, CC compared to search term-identified websites were more likely to provide guidance based on the result of the test (*p*<0.001) and resources for genetic counseling (*p*<0.001).

**Table 2:** Comparison of web-based information on cell-free DNA (cfDNA) prenatal screening provided by commercial company and search term identified websites.

Information on cfDNA	Commercial Company (N=11) N (%)	Search Term- Identified (N=13) N (%)	*P* Value
Ancillary resources	11 (100)	13 (100)	1.000
Mechanism of the test	10 (90.9)	13 (100)	0.458
Screening for trisomy 21	11 (100)	5 (38.5)	0.002*
Screening for trisomy 18	11 (100)	4 (30.8)	<0.001*
Screening for trisomy 13	11 (100)	4 (30.8)	<0.001*
Screening for sex chromosome aneuploidies	11 (100)	1 (7.7)	<0.001*
Screening for microdeletions	8 (72.7)	1 (7.7)	0.002*
Screening for triploidy	1 (9.1)	0 (0)	0.458
Screening for other trisomy conditions	2 (18.2)	0 (0)	0.199
Fetal sex determination	11 (100)	12 (92.3)	0.542
Ease of interpretation of test result	11 (100)	9 (69.2)	0.067
Comments on validity of cfDNA	9 (81.8)	12 (92.3)	0.353
Sensitivity/specificity for general performance	6 (54.5)	1 (7.7)	0.017*
Sensitivity/specificity for fetal sex determination	3 (27.3)	3 (23.1)	0.351
Limitations for general application	3 (27.3)	2 (15.4)	0.303
Limitations for fetal sex determination	2 (18.2)	2 (15.4)	0.404
Benefits	11 (100)	7 (53.8)	0.013*
Risks	10 (90.9)	2 (15.4)	<0.001*
Application with multiple pregnancy	9 (81.8)	0 (0)	<0.001*
Guidance based on the result of the test	11 (100)	4 (30.8)	<0.001*
Resources for genetic counseling	9 (81.8)	0 (0)	<0.001*

*statistically significant with p < 0.05.

CC and search term-identified websites offered ancillary resources (*p*=1.000) equally and both sources were equally likely to explain the mechanism (*p*=0.458) and validity of cfDNA (*p*=0.353). Both CC and search term identified websites reported that cfDNA can be used for fetal sex determination with similar frequency (*p*=0.542). More CCs (100%) reported ease of test result interpretation than search term-identified websites (69%), but this difference was not statistically significant (*p*=0.067). Few CC or search term-identified websites discussed using cfDNA to screen for triploidy or other trisomy conditions, sensitivity and specificity of cfDNA for fetal sex determination, and limitations of cfDNA for general application and fetal sex determination.

## 4 Discussion

This study identified and characterized the websites that expectant parents are most likely to access when seeking information about cfDNA for fetal sex determination. Most of the identified websites represent commercial testing companies and are authored by their representatives. Other sites represent non-professional entities and are authored by non-professionals. These findings support our hypothesis that most information accessed by expectant parents is predominantly from non-professional organizations. Furthermore, the information provided on CC and search term-identified websites highlights the importance of the source of information. CCs were more likely to promote cfDNA for pregnancies of multiples, emphasize that it is easy to interpret cfDNA results, and state higher number of fetal conditions that cfDNA can detect than search term-identified websites.

It is important to acknowledge that this new screening technology offers many advantages, including early use in pregnancy, being less invasive than traditional prenatal diagnostic testing methods like chorionic villus sampling (CVS) and amniocentesis, use for detecting pregnancies at high risk of certain medical conditions, and ability to predict fetal sex (2, 3). cfDNA is not intended solely to predict fetal sex, but sex can be critical in certain conditions where sex influences the clinical course and management, such as X-linked conditions, sex limited conditions (e.g., conditions that affect organs limited to typical male or female development) and differences of sex development (DSD), such as congenital adrenal hyperplasia (2, 7, 26–28). Despite numerous advantages, CC websites primarily advertise cfDNA as a “baby gender mentor,” yet are less likely to provide information about risks associated with cfDNA testing. For predicted fetal sex determination, it is important to note that cfDNA predicts the fetus to be “male” if Y chromosome material is detected (2, 3, 26). However, this can be misleading in DSD and in cases of genotype-phenotype discordance, such as when the US findings are consistent with a different fetal sex than cfDNA predicted fetal sex. The discordant results can lead to further investigation, referral to specialized centers, clinical dilemma and uncertainty, and information overload (29). This unexpected or unwanted detection of an abnormality was the reason for one of the claims for the class-action lawsuit filed against the test supplier (18, 21, 22, 30).

Potential negative consequences should not be surprising, given that the cfDNA test was never subjected to any evaluation by any regulatory authority, including the FDA. In some situations, families undergo invasive tests, and psychological and emotional turmoil unnecessarily because of cfDNA (14, 31). For example, Richardson and colleagues described a case where cfDNA prenatal screening did not detect Y chromosomal material, thus predicting female (XX) fetal sex, but US showed a male-typical phenotype. Subsequent invasive diagnostic testing revealed translocation of Y chromosome material, including *SRY*, onto the X chromosome. The baby was born as a healthy boy. In another case, cfDNA prenatal screening demonstrated an increased risk for Klinefelter syndrome or 47XXY due to an increase in X chromosomal material detected on analysis, but US showed a female-typical phenotype. Further invasive diagnostic tests were pursued and clarified that a fragment of Y chromosome material without *SRY* was present, explaining the female-typical phenotype. Interestingly, this chromosomal finding was maternally inherited, with the mother found to have the same genotype as the fetus. A healthy baby girl was born at term (13). Alternatively, there was also a case where cfDNA detected Y chromosome material contributed by a vanishing twin, while the viable twin was female in sex. These cases illustrate situations where cfDNA prenatal screening was the reason for additional invasive diagnostic testing, exposing both mothers and fetuses to otherwise unnecessary risks. Also, because of cfDNA, babies, and mothers in some cases, are noted in medical charts as having genetic abnormalities or diagnoses in their medical record without clear clinical significance or health benefit.

One of the underlying reasons for expectant parents’ unpreparedness, confusion, psychological distress and other negative experiences with cfDNA is the information sources, content, and mode of information transfer (14, 32, 33). The medical community has recognized that attention to consumer education and assessment of the accuracy of information provided by CCs are inadequate (6, 17). As expected, a significant number of expectant parents access information about cfDNA through website types that are not generated by professional organizations (14, 26). It has been acknowledged across multiple studies that publicly available information can easily be inaccurate or of low-quality, and that there is need to ensure that information, accessed by patients, is accurate and based on evidence (6, 7, 14, 26). In addition to misinformation, Cakar and colleagues also demonstrated that the mode of information transfer, in itself, can be a significant source of anxiety, showing that most cfDNA test consumers (63%) accessed information from non-professional sources, and when compared across different sources of information, receiving information from a professional source was associated with the lowest level of anxiety compared to receiving information from non-professional sources (14). Further, it is alarming that over 15% physicians do not offer cfDNA prenatal screening, and among those who offer, almost 48% do not provide in-depth pretest counseling and refer to a genetic counselor or MFM instead (34). Farrell and colleagues demonstrated that a significant portion of physicians reported their sources of information to be from a representative of a commercial laboratory (29%), or literature, website or other educational opportunity hosted by a commercial laboratory (27%) (34).

Our team has encountered expectant parents who were referred to our multidisciplinary DSD program for further investigations due to discordant results between cfDNA predicted fetal sex and US findings, or other prenatal suspicion of DSD such as atypical genitalia on US with concern for sex chromosome DSD on cfDNA (35, 36). These prenatal results represented both false positives (e.g., Y chromosome material detected due to vanishing twin) and true positives (e.g. Y chromosome material detected due to complete androgen insensitivity syndrome) (36). Many of these parents were experiencing distress, confusion, anxiety, and depression (35). Two mothers were even considering pregnancy termination. One mother reported that she had a “breakdown” when she received diagnosis, and she was considering pregnancy termination and having suicidal thoughts (35). Concerningly, it has been reported that 6.2% of women will terminate their pregnancy, based on the positive cfDNA screening result, without confirming the diagnosis with a conventional and diagnostic invasive test, such as CVS or amniocentesis (37). Additionally, these parents have reported to the authors of this study that discordant results and subsequent prenatal diagnoses cause disappointment, ongoing conflict between partners, and difficulty of bonding with their unborn babies.

Although cfDNA is already widely available by CCs in the US, increased standardized clinical use, parent/patient education, and physician awareness are urgently needed. It is imperative that academic medical centers, especially DSD programs, develop patient education information that is easily accessible. Clinicians also need to be aware and ready to guide expectant parents through their decision-making process, especially at times when results between cfDNA and standard screening tests are discordant (7, 12, 26, 38–42).

This is the first and only study to our knowledge to review currently available online information about cfDNA prenatal screening. However, the study has several limitations. Only one search engine, Google, was used, although is the most widely-used search engine and captures most search activities. Information types that were not web-based were not assessed (e.g., pamphlets, magazines, brochures). Language was restricted to English, hence information sources in other languages were not assessed. This study also did not assess the quality of the information (e.g., readability, understandability, accuracy, completeness, actionability) as the purpose of the study was to assess availability and content of information.

## 5 Conclusion

This study explored the availability and content of websites about cfDNA as a tool for prenatal screening. The findings of the study show that most web-based information about cfDNA that is accessed by expectant parents are from non-professional organizations and there is a significant discrepancy between information provided by CC and search term-identified websites, especially about performance of cfDNA screening. Expectant parents may thus be inadequately informed about the possibility of discordant results, such as prenatal fetal abnormities like DSD, between cfDNA and standard screening methods. To minimize such negative consequences, standards for clinical use, parent/patient education and physician awareness are urgently needed.

## Data availability statement

The raw data supporting the conclusions of this article will be made available by the authors, without undue reservation.

## Author contributions

SK, IR, JH, and EJ contributed to conception and design of the study. SK, EF, and UN organized the database. SK and IR performed the statistical analysis. SK wrote the first draft of the manuscript. SK, IR, and EJ wrote sections of the manuscript. All authors contributed to the article and approved the submitted version.
